# Mobility care in nursing homes: development and psychometric evaluation of the kinaesthetics competence self-evaluation (KCSE) scale

**DOI:** 10.1186/s12912-017-0257-8

**Published:** 2017-11-21

**Authors:** Heidrun Gattinger, Beate Senn, Virpi Hantikainen, Sascha Köpke, Stefan Ott, Helena Leino-Kilpi

**Affiliations:** 1 Finnish Doctoral Programme in Nursing Science, Department of Nursing Science, University of Turku, Turku, Finland; 2 Institute of Applied Nursing Sciences, University of Applied Sciences FHS St. Gallen, Rosenbergstrasse 59, Postfach, 9001 St. Gallen, Switzerland; 3 Institute of Applied Nursing Sciences, University of Applied Sciences FHS St.Gallen, St. Gallen, Switzerland; 4 Research Affiliate Sydney Nursing School, University of Sydney, Sydney, Australia; 5 Adjunct Professor Department of Nursing Science, University of Turku, Turku, Finland; 6 Research Affiliate Institute of Applied Nursing Sciences, University of Applied Sciences FHS St. Gallen, St. Gallen, Switzerland; 7 Institute for Social Medicine and Epidemiology, Nursing Research Unit, University of Lübeck, Lübeck, Germany; 8 University of Applied Sciences FHS St. Gallen, St. Gallen, Switzerland; 9 Department of Nursing Science, University of Turku, Turku, Finland; 10 Turku University Hospital, Turku, Finland

**Keywords:** Kinaesthetics, Mobility limitations, Clinical competence, Educational measurement, Nursing

## Abstract

**Background:**

Impaired mobility is a prevalent condition among care-dependent persons living in nursing homes. Therefore, competence development of nursing staff in mobility care is important. This study aimed to develop and initially test the Kinaesthetics Competence Self-Evaluation (KCSE) scale for assessing nursing staff’s competence in mobility care.

**Methods:**

The KCSE scale was developed based on an analysis of the concept of nurses’ competence in kinaesthetics. Kinaesthetics is a training concept that provides theory and practice about movement foundations that comprise activities of daily living. The scale contains 28 items and four subscales (attitude, dynamic state, knowledge and skills). Content validity was assessed by determining the content validity index within two expert panels. Internal consistency and construct validity were tested within a cross-sectional study in three nursing homes in the German-speaking region of Switzerland between September and November 2015.

**Results:**

The content validity index for the entire scale was good (0.93). Based on a sample of nursing staff (*n* = 180) the internal consistency results were good for the whole scale (Cronbach’s alpha = 0.91) and for the subscales knowledge and skills (α = 0.91, 0.86), acceptable for the subscale attitude (α = 0.63) and weak for the subscale dynamic state (α = 0.54). Most items showed acceptable inter-item and item-total correlations. Based on the exploratory factor analysis, four factors explaining 52% of the variance were extracted.

**Conclusion:**

The newly developed KCSE scale is a promising instrument for measuring nursing staff’s attitude, dynamic state, knowledge, and skills in mobility care based on kinaesthetics. Despite the need for further psychometric evaluation, the KCSE scale can be used in clinical practice to evaluate competence in mobility care based on kinaesthetics and to identify educational needs for nursing staff.

## Background

People living in nursing homes and residential facilities often require assistance with mobility including walking, transferring, and bed mobility, as well as with movements needed to accomplish activities of daily living (such as dressing, personal hygiene, or toileting) [1]. In this article, we will refer to this kind of assistance as mobility care [2]. Mobility impairment can result from age-related slowly progressive functional loss due to neuromuscular, cognitive or sensory decline [3] or can occur suddenly after an acute event such as a stroke [4].

Mobility is necessary for participation in meaningful social, cultural, and physical activities. Impaired mobility restrict participation in social activity leading to isolation and loneliness [5]. Furthermore, loss of mobility is associated with a persistent decline in function and physical activity and is a risk factor for pressure ulcers, falls, urinary incontinence, and malnutrition [6]. Because mobility contributes to the residents’ health and quality of life, it is important to maintain and enhance this mobility [7]. Therefore, nurses (registered and licensed practical nurses) and other care team members who work in direct care (nurse assistants and nursing aides) – hereinafter referred to as nursing staff – should have the knowledge and skills that allow them to provide high quality mobility care [8]. Nursing staff is required to understand the residents’ mobility capacities and to use mobility enhancing strategies and methods encouraging residents, where possible, to move to their highest level of independence [9]. Different nursing staff training programs have been tested in order to reach these goals; for example, an educational intervention in natural mobility [10] and a person-centered approach with mobility enhancing strategies, which include how to interact and communicate with residents and how to promote the correct biomechanics [11].

Another comprehensive training concept is kinaesthetics, it provides theory and practice about movement foundations that comprise activities of daily living. The kinaesthetics approach includes person-centeredness, personalized interaction and communication as well as appropriate support of functional mobility [12, 13]. In kinaesthetics training, participants learn to understand human movement and a mobility impaired persons’ possibilities to participate in activity of daily living based on the six concepts interaction, functional anatomy, human movement, human functions, effort, and environment [12, 13].

Competence criteria relevant to provide high quality mobility care are represented in knowledge, skills, attitude and a dynamic state. Knowledge comprises theoretical understanding of safe and optimal handling during movement support of a mobility impaired person [11, 14, 15]. Skills include effective communication, attentive interaction, ability to support a care-dependent person’s movement in an optimal way, nursing staff member’s ability to adapt their own movement, and adaptation of the environment in order to enhance the mobility impaired person’s individual way of moving [15–19]. Attitude includes interest and openness toward the care-dependent person and a commitment to personal development [10, 15, 17, 18]. Dynamic state includes analysis and reflection of mobility support situations as well as provision of learning situations [15, 17, 18, 20].

However, high quality mobility care is not necessarily a clearly recognized nursing remit [21] and the training and support that nursing staff need regarding mobility care to adequately fulfil their role are lacking [8, 22]. In order to identify the educational needs regarding nursing staff’s knowledge, skills, attitude, and dynamic state in mobility care and to guide further practical and theoretical training, an assessment of nursing staff competence in mobility care is essential.

Former self-evaluation instruments were developed in order to evaluate specific training concepts, e.g. natural mobility [10]. Five instruments were identified that assess nursing staff’s knowledge and skills regarding learned principles [11, 23–26]. Instruments developed by Betschon et al. (2011), Kindblom-Rising et al. (2011) and Kneafsey and Haigh (2009) include questions for assessing participants’ attitude and motivation and perception of their role in mobility rehabilitation. Although, most of these instruments underwent preliminary testing regarding face and/or content validity with experts and/or nursing staff [11, 24–26], only few have reported additional psychometric testing [10, 26]. No self-evaluation instrument was identified that includes all areas of nursing staff’s competence in mobility care based on kinaesthetics.

Therefore, the goal of the current study was to develop a valid and reliable self-evaluation instrument in order to determine nursing staff competence in mobility based on kinaesthetics [15]. The present article summarizes the development of the Kinaesthetics Competence Self-Evaluation (KCSE) scale, the testing of content validity and the analysis of internal consistency and construct validity of the scale.

## Methods

### Construction of the KCSE scale

First, a blueprint with item pool and response scale was established based on a concept development study [15]. In an iterative process, the items and response scale were refined between January and June 2015. Subsequently, a scale with four categories (knowledge, skills, attitude, and dynamic state) and 28 items was assessed for content validity. Content validity was evaluated first with a panel of nine experts with kinaesthetics trainer certificates and varied experience in health care (Table 1). First, the expert panel rated each item on a 4-point Likert scale (1 = not relevant, 2 = somewhat relevant, 3 = quite relevant, 4 = highly relevant). Second, the experts evaluated the clarity of items. Finally, the experts were asked for further comments / suggestions to improve the scale. Items with inter-rater agreement for relevant and somewhat relevant items of ≥0.78 were accepted for inclusion in the scale [27]. Based on these results, two items were deleted, 20 items reworded, and two new items added. The revised scale was re-evaluated with a second panel of five experts (Table 1) using the same procedure.

**Table 1:** Sociodemographic characteristics of experts in both panels

Characteristics	Content validity testing	
	1. Expert panel (*n* = 9)	2. Expert panel (*n* = 5)
Age: mean (SD)		
In years	51.4 (4.25)	51.4 (2.51)
Working experience with kinaesthetics: mean (SD)		
In years	19.9 (6.74)	19.6 (2.97)
Gender (n, %)		
Female	7 (78%)	3 (60%)
Male	2 (22%)	2 (40%)
Nationality (n, %)		
Swiss	7 (78%)	4 (80%)
German	2 (22%)	1 (20%)
Profession (n, %)		
Registered nurse	5 (56%)	5 (100%)
Licensed practical nurse	2 (22%)	0
Physiotherapist	1 (11%)	0
Speech therapist	1 (11%)	0
Kinaesthetics training ^Δ^ (n, %)		
Trainer level 1	0	1 (20%)
Trainer level 2	0	0
Trainer level 3	6 (67%)	3 (60%)
Train the trainer	3 (33%)	1 (20%)
Main place of work* (n, %)		
Long-term care for elderly	6 (67%)	5 (100%)
Home care	3 (33%)	0
Hospital care	2 (22%)	1 (20%)
Long-term care for disabled persons	5 (56%)	1 (20%)

*SD* standard deviation; ^**Δ**^ According to European Kinaesthetics Association, trainer levels are based upon each other and train the trainer level is the highest level [24]; * includes double entries (added up to more than 100%)

In the revised scale, the item content validity index (I-CVI) [27] was 1.0 for 18 items and 0.8 for 10 items, and the content validity index for the entire scale (S-CVI) [27] with 28 items was 0.93.

In order to assess comprehensibility and usability, the questionnaire was pilot tested with a group of nursing staff (*n* = 6; two registered nurses, two licensed practical nurses, and two nursing aides with a mean work experience in long-term care of 8 years, SD 7.4), working in two medium-sized (around 80 beds) nursing homes. The participants evaluated the clarity of instruction, item wording, and the questions about sociodemographic characteristics and background variables and, if they had any concerns, their suggestion for revision. A specification in one sociodemographic question and a further explanation in the instruction (to not set the cross between boxes) were made based on the participants’ responses.

### Description of the final KCSE scale

**Fig. 1:**
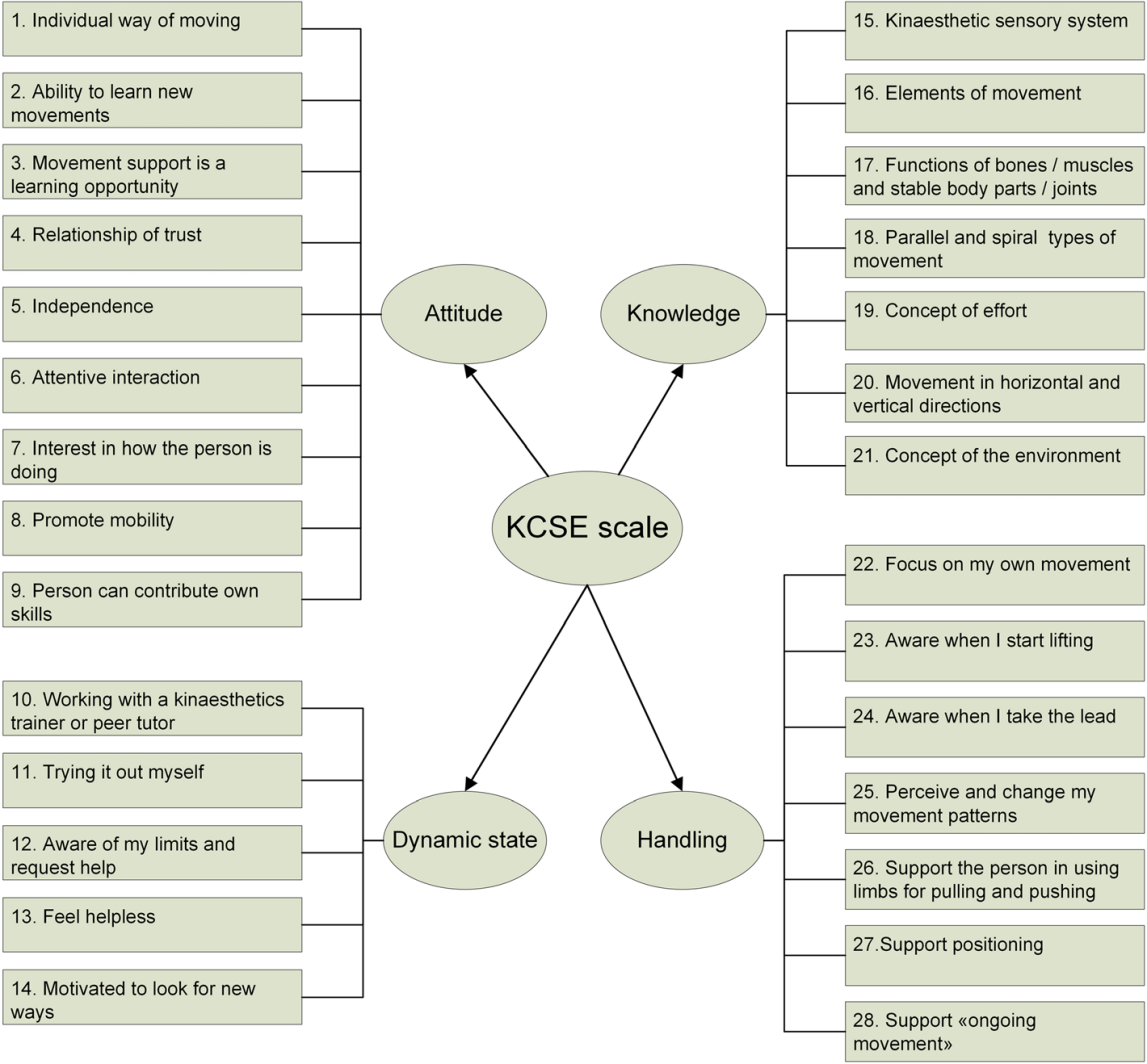
Structure of the KCSE scale

The final KCSE scale comprises 28 items assessing nursing staff’s *attitude* (9 items) and *dynamic state* (5 items) about mobility enhancing care in terms of kinaesthetics, their *knowledge* of the kinaesthetics concept (7 items), and their self-perceived use of the kinaesthetics principles (=*skills*) (7 items) (Fig. 1). Items have four response options in terms of agreement (disagree, somewhat agree, agree, strongly agree), frequency (never, sometimes, almost every time, every time), and level of quality (not at all, somewhat, good, very good). Single items score between 1 and 4, with higher scores indicating higher self-evaluated competence in mobility care based on kinaesthetics. The instrument has been developed in German [28].

### Psychometric evaluation of the KCSE scale

Psychometric evaluation of the KCSE scale included testing of reliability (internal consistency) and construct validity [29] within a cross-sectional study.

### Settings, sample, and data collection

The study was conducted in three nursing homes in the German-speaking part of Switzerland between September and November 2015. The nursing homes were purposefully selected based on the following criteria: medium-sized nursing home (between 50 and 100 beds), private or public, not exclusively providing care for demented persons and a minimum of half the nursing staff members passed a kinaesthetics training. These criteria are representative of nursing home facilities in the German-speaking region of Switzerland [30].The KCSE scale was handed out to all German-speaking nursing staff (i.e., registered nurses, licenced practical nurses, nursing aides and nursing students) working in direct care by the head nurse. Data was collected over a 4-week period, and a reminder was sent to the nursing homes after another two weeks. The completed KCSE scale was returned in a sealed envelope to boxes that were placed in the participating wards.

### Data analysis

The data was analyzed using SPSS 22 (IBM Corp, 2013). Item level, subscale level, and total-scale level analyses were conducted using descriptive statistics (frequencies, means, and standard deviation). For the KCSE subscales, mean scores were calculated (range 1–4). The total score was calculated by summing the mean scores of the subscales (range 4–16).

Internal consistency was calculated using Cronbach’s alpha coefficients. Cronbach’s alpha coefficients less than 0.60 are considered low, indicating limited instrument consistency [31]. Item analyses were performed by computing the corrected item-total correlation for the items in the subscales and an inter-item correlation. Item-total correlations of at least 0.20 were regarded as acceptable [32], as were inter-item correlations of *r* > 0.20 and <0.70 [32, 33].

The construct validity of the KCSE scale was investigated with exploratory factor analysis in order to explore the underlying structure of the 28-item KCSE scale. To confirm the adequacy of the sample size for factor analysis, the variable to subject ratio was calculated; 1:5 was considered the minimum and 1:10 sufficient [34]. Prior to performing factor analyses, the suitability of the data was assessed using the Kaiser-Meyer-Olkin (KMO) criterion with a recommended value of 0.5 or above and Bartlett’s test of sphericity with a *p*-value below 0.05 [35]. Principal component analysis (extraction method) was conducted using direct oblimin rotation in order to account for correlations between factors. Eigenvalues greater than one (Kaiser criterion) and the scree plot were used to determine the number of factors [35].

## Results

### Sample characteristics

From the total sample of 214 nursing staff members, 180 (=n) returned the questionnaire (84%) (Table 2).

**Table 2:** Sociodemographic and professional characteristics of participating nursing staff (*n* = 180)

Characteristics	n	%	Mean (SD)	Range
Age (years)	175		41.8 (13.23)	15–64
Length of experience in nursing home care (years)	175		12.9 (9.99)	0–37
Length of working in the current institution (years)	176		8.3 (7.93)	0–36
Gender				
Female	159	88%		
Male	21	12%		
Educational level				
Registered nurse (BSN, Diploma)	58	32%		
Licensed practical nurse	30	17%		
Nurse assistant	80	44%		
Nursing student	7	4%		
Missing data	5	3%		
Level of employment				
80–100%	100	56%		
50–70%	49	27%		
20–40%	27	15%		
Missing data	4	2%		
Kinaesthetics training [32]				
No training	16	9%		
Basic training course	69	38%		
Advanced training course	66	36%		
Peer tutoring training	23	13%		
Trainer education	5	3%		
Missing data	1	1%		

### Scale descriptive and internal consistency results

Nursing staff’s mean sum score (range 4–16) on the KCSE scale was 13.0 (SD 1.437). Mean scores that participants attained in the subscales (range 1–4) ranged from 3.0 to 3.6 (Table 3).

**Table 3:** Descriptive and internal consistency results of the KCSE scale

Dimensions of KCSE scale	n	Score range	Mean (SD)	No of items	Cron-bach’s alpha	Item-total correlation r > 0.20	Inter-item correlations r > 0.20 & *r* < 0.70
Attitude	174	2.8–4	3.6 (0.27)	9	0.63	8/9, 89%	12/36, 33%
Dynamic state	165	2.4–4	3.4 (0.40)	5	0.54	4/5, 80%	6/10, 60%
Knowledge	172	1–4	3.0 (0.59)	7	0.91	7/7, 100%	20/21, 95%
Skills	170	1.1–4	3.0 (0.50)	7	0.86	7/7, 100%	21/21, 100%
Total scale	150	8.2–16.0	13.0 (1.44)	28	0.91	24/28, 86%	

The reliability of the scale was assessed by Cronbach’s alpha and item analysis including item-total and inter-item correlations. The entire KCSE scale shows high internal consistency with a Cronbach’s alpha coefficient of 0.91. For the subscale levels, the coefficients were 0.63 for *attitude*, 0.54 for *dynamic state*, 0.91 for *knowledge*, and 0.86 for *skills* (Table 3). Regarding the item analysis, 86% of all items showed higher item-total correlations than the criteria set (*r* > 0.20) (Table 3). The lowest correlations were obtained for item 1 (individual way of moving), item 4 (relationship of trust), item 12 (aware of my limits and seek help), and item 13 (feel helpless) (Table 4). The aimed inter-item correlations of r > 0.20 and <0.70 was reached for 33–100% of the subscale items (Table 3).

**Table 4:** Corrected item-total correlations and factor analysis with direct oblimin rotation of the KCSE scale

Item No	Abbreviated item ^a^	Corrected item-total correlation	Rotated Factor Loadings			
			F1	F2	F3	F4
*Attitude*						
1	Individual way of moving	.075	−.010	.013	**.594**	−.109
2	Ability to learn new movements	.363	.349	.012	**.614**	−.333
3	Movement support is a learning opportunity	.367	.304	.175	**.603**	−.141
4	Relationship of trust	.139	.059	.112	**.585**	.270
5	Independence	.248	.161	.233	**.549**	.013
6	Attentive interaction	.223	.125	**.609**	−.209	−.078
7	Interest in how the person in doing	.335	.171	**.661**	.281	−.032
8	Promote mobility	.430	.289	**.694**	.240	.096
9	Person can contribute own skills	.357	.241	**.529**	.281	.004
*Dynamic state*						
10	Working with a kinaesthetics trainer or peer tutor	.487	.454	.423	.065	**−.476**
11	Trying it out myself	.487	.428	**.608**	−.065	−.317
12	Aware of my limits and request help	.147	.019	.417	.078	**−.630**
13	Feel helpless	.175	.245	.148	−.050	**.609**
14	Motivated to look for new ways	.414	.307	**.531**	.182	−.346
*Knowledge*						
15	Knowledge about kinaesthetics sensory system	.767	**.829**	.337	.203	.133
16	Knowledge about elements of movement	.679	**.776**	.218	.150	.065
17	Knowledge about function of bones and muscles and stable body parts and joints	.637	**.764**	.088	.179	−.036
18	Knowledge about parallel and spiral types of movement	.587	**.752**	.013	.035	−.182
19	Knowledge about the concept of effort	.645	**.787**	.125	−.018	−.102
20	Knowledge about characteristics of movement in horizontal and vertical directions	.740	**.794**	.286	.248	−.088
21	Knowledge about the concept of environment	.653	**.775**	.086	.188	−.137
*Skills*						
22	Focus on my own movement	.679	**.664**	.512	.175	−.006
23	Aware when I start lifting	.609	**.647**	.326	.201	.063
24	Aware when I take the lead	.646	**.747**	.254	.128	.209
25	Perceive and specifically change my movement patterns	.680	**.693**	.434	.196	.071
26	Support the person in using limbs for pulling and pushing	.612	**.626**	.369	.238	−.052
27	Support positioning	.659	**.673**	.454	.148	.191
28	Support “ongoing movement”	.596	**.670**	.366	−.090	.034

^a^English language translation of the original German items

F1 first factor, F2 second factor, F3 third factor, F4 fourth factor

Bold figures indicate the highest loading of an item on its theoretical component

### Structure of the instrument

An exploratory factor analysis was used to explore the underlying factors of the newly developed KCSE scale. The Kaiser-Meyer-Olkin measure verified the sampling adequacy for the analysis (KMO = 0.89). Bartlett’s test was significant (*p* < 0.001) and indicated that correlations between items were sufficiently large for exploratory factor analysis [35]. An initial analysis was run to obtain eigenvalues for each component of the data. Six components had eigenvalues over Kaiser’s criterion of 1 and in combination explained 59.92% of the variance.

Solutions for six, five, and four factors were each examined using direct oblimin rotations of the factor loading matrix. The four-factor solution, which explained 52.37% of the variance, was chosen because the scree plot showed inflexions that justified retaining the four components and all items met the criterion of having a primary factor loading of 0.4 or above [35]. Table 4 shows the factor loadings after rotation.

**Fig. 2:**
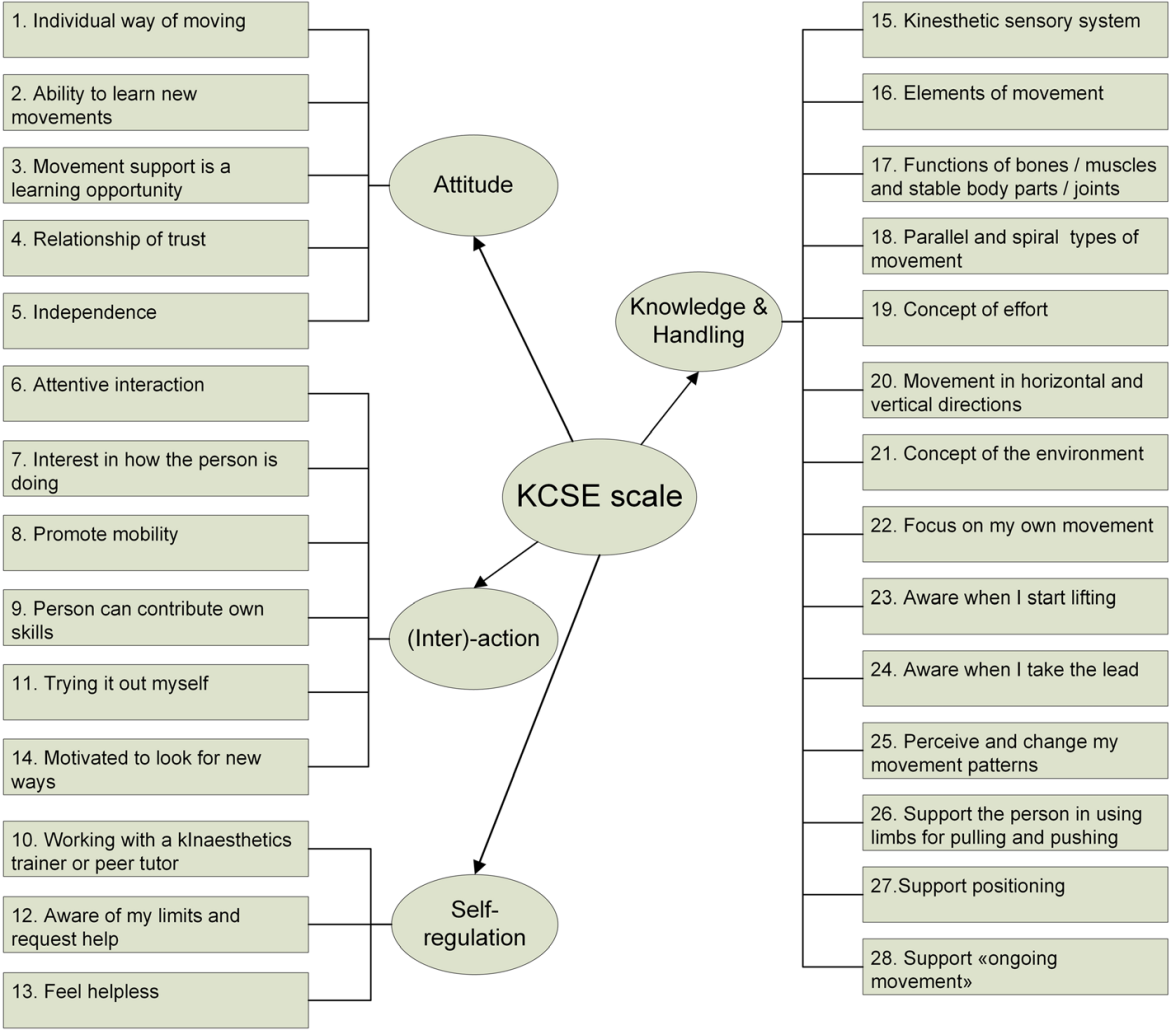
Structure of the KCSE scale based on explorative factor analysis

Based on the explorative factor analysis, another structure in the dimensions of the KCSE scale emerged (Fig. 2). All items from the *knowledge* (items 15–21) and *skills* (items 22–28) subscales loaded on the first factor. The second factor includes items from the *attitude* (items 6–9) and *dynamic state* (item 11, 14) subscales. The third factor captures five items from the *attitude* (items 1–5) subscale. The fourth factor includes three items from the *dynamic state* (items 10, 12, 13) subscale. Labeling of the factors is based on the theoretical underpinning of the study and reflects the theme of the factor items themselves: F1 knowledge & skills (14 items), F2 (inter-) action (6 items), F3 attitude (5 items) and F4 self-regulation (3 items) (Fig. 2).

## Discussion

The aim of this study was to develop and test the Kinaesthetics Competence Self-Evaluation (KCSE) scale. For the first time the relevant competence areas – knowledge, skills, attitude, and dynamic state – for mobility care based on kinaesthetics are measurable in one instrument. In the KCSE scale all domains are equally important, which is reflected in the composition of the sum score (mean subscale scores add up to a sum score). Other self-evaluation instruments used in this field differ in their foci, e.g. the instrument developed by Kindblom-Rising et al. (2011) assesses nursing staff’s movement and body awareness, attitude to the patient, to oneself and to work as well as reported behaviour. The questionnaire developed by Kneafsey et al. (2012) focuses on safety aspects of mobility care. Based on the content of the instruments it can be assumed that there are similarities between the different training approaches, e.g. the requirement to understand a care-dependent persons’ mobility capacity [11] or the awareness of one’s own movement [10]. However, it would be of great interest to condense elements from different training approaches to establish central elements reflecting high quality mobility care.

The new developed KCSE scale obtained appropriate values for internal consistency for the subscales *attitude*, *knowledge* and *skills*. The subscale *dynamic state* showed internal consistency values below the recommended 0.60. The inter-item correlations varied, but most items of the subscales *dynamic state*, *knowledge* and *skills* showed acceptable values. However, 67% of the items from the subscale *attitude* did not reach the criterion for inter-item correlation of *r* > 0.20 and <0.70. Values below 0.20 may not contribute significantly to the measurement of the concept, and those above 0.70 may be capturing only a small bandwidth of the construct [33]. Item-total correlations revealed four problematic items: item 1 (individual way of moving), item 4 (relationship of trust), item 12 (aware of my limits and seek help) and item 13 (feel helpless) showed item-total correlations below 0.2. These items may not be sensitive enough to assess nursing staff’s attitude and dynamic state related to mobility care. There is clearly a need to further assess the sensitivity of these items. The scale descriptives show that responses are not evenly distributed, but are skewed toward the upper end of the scale. Thus, a ceiling effect is possible and this means that it is difficult to detect any improvement, or distinguish among various grades of excellence [32]. In this study, more than 90% of the participants had passed a kinaesthetics training and the results indicate that the participants are confident with the ideas of the training. Further testing of the KCSE scale should be conducted in other samples (e.g. nursing staff working in hospital or home care), before considering another response category at the upper end of the scale.

In this study an exploratory factor analysis was conducted in order to explore the structure of the new measurement [34], showing that four factors explain 52% of the variance. These four factors show a logical connection to the theoretical framework of the instrument, although they differ slightly from the dimensions used during the design of the instrument. Items from the subscale *knowledge* and *skills* joined together to factor 1. This is also theoretically plausible as these items reflect the knowledge and the application of the kinaesthetics concept system [36] and, therefore, are closely related to each other. The items of the subscales *attitude* and *dynamic state* are split into three different factors (factor 2, 3, and 4). There is a need for further analysis of the theoretical structure of the scale. The conceptual framework of competence in mobility care based on kinaesthetics is founded on literature and experts’ judgement [15]. As this framework is a new development, it should be verified by further research.

### Limitations of the study

An inherent limitation of any study in which participants are volunteers is the potential for non-response bias. Although the response rate was high in this study (84%), non-respondent nursing staff might have responded differently to the KCSE scale. Furthermore, the sample included groups that were small (nursing students or nurses with kinaesthetics trainer education). Therefore, the reliability and validity results may not apply for these groups. The KCSE scale needs further testing in a larger and more diverse sample. Another limitation of our study is its cross-sectional design. Therefore, we are not yet able to establish the instrument’s sensitivity to change in competence development.

### Practical implications

The KCSE scale is a short (28 items) and easy to be administered instrument that can be used to assess nursing staff’s attitude, dynamic state, knowledge and self-perceived skills in mobility care based on kinaesthetics. It can be applied for RNs, LPNs, nurse assistants and nursing aides with and without kinaesthetics training. The instrument has several potential applications for health care institutions aiming to foster high quality mobility care. First, the assessment of current mobility care practice from a self-evaluated perspective may highlight competencies that require training at an individual or institutional level. Second, the instrument may be used for reflective application, in order to facilitate nursing staff’s awareness of possible discrepancies between their attitude, dynamic state, knowledge, and skills. Third, this assessment can help kinaesthetics trainers tailor the content and teaching strategies of training courses. Fourth, the self-evaluated competence can be compared with observed competence in kinaesthetics [37] to reinforce or dispel nursing staff‘s perceptions.

### Future research implications

Several recommendations can be applied to the instrument to improve its validity and reliability and its relevance in other contexts. Based on further research in other samples, the theoretical structure of the instrument should be further analyzed and items and scale modification should be considered. The instrument should be tested within a confirmatory factor analysis and an adequate sample size [38]. In order to investigate the instrument’s sensitivity to change, longitudinal studies should be conducted. Considering that supporting the mobility of care-dependent persons is the responsibility of the whole nursing team, it is of interest to investigate differences in larger and more diverse groups (e.g. nurse students or nursing aids) and to determine influencing factors (e.g. work experience). Subsequently, studies investigating the impact of higher levels of competence in kinaesthetics on patient outcomes (e.g. on functional mobility) should be conducted.

## Conclusion

The newly developed KCSE scale is a promising instrument that can be used for measuring nursing staff’s attitude, dynamic state, knowledge, and skills in mobility care based on kinaesthetics. The first psychometric testing showed good scale content validity (S-CVI =0.93) and acceptable reliability and validity results when administered by a sample of nursing staff in nursing home care.

Despite the need for further psychometric evaluation, the scale’s application in clinical practice may be useful for identifying nursing staff’s educational needs and evaluating personal growth in competence in mobility care based on kinaesthetics.

## Abbreviations

CVI: Content validity index
I-CVI: Item content validity index
KCSE: Kinaesthetics competence self-evaluation
S-CVI: Scale content validity index
